# Pulsed laser-assisted additive manufacturing of Ti-6Al-4V for in-situ grain refinement

**DOI:** 10.1038/s41598-022-26758-y

**Published:** 2022-12-23

**Authors:** Hansol Yoon, Peipei Liu, Yejun Park, Gwanghyo Choi, Pyuck-Pa Choi, Hoon Sohn

**Affiliations:** 1 Global R&D Center, SQ Engineering, Seoul, 05818 Republic of Korea; 2 Department of Civil and Environmental Engineering, Korea Advanced Institute of Science and Technology (KAIST), Daejeon, 34141 Republic of Korea; 3 Present Address: Tomocube, Daejeon, 34109 Republic of Korea; 4 Center for 3D Printing Nondestructive Testing, KAIST, Daejeon, 34141 Republic of Korea; 5 Department of Materials Science and Engineering, KAIST, Daejeon, 34141 Republic of Korea; 6 Present Address: Analysis Solution Center, SK Innovation, 34124 Daejeon, Republic of Korea

**Keywords:** Design, synthesis and processing, Laser material processing, Characterization and analytical techniques

## Abstract

Metal additive manufacturing (AM) enables rapid customization of complex parts. However, it leads to forming of columnar grain structures which give the AM parts anisotropic properties. In this study, we propose a pulsed laser-assisted AM (PLAAM) technique for in-situ grain refinement of Ti-6Al-4V parts. A nanosecond pulsed laser was focused onto a melt pool to generate a favorable environment for the promotion of fine equiaxed grains. The PLAAM technique provided an average prior-β grain size of 549.6 μm, compared to that of 1297 μm provided by the conventional AM technique. Moreover, the maximum value of multiples of uniform distribution of the β phase decreased from 16 to 7.7 when using the PLAAM technique, which indicates a weakened crystallographic texture. These changes confirm that the proposed PLAAM technique promotes finer and more equiaxed prior-β grains. Furthermore, because the proposed technique is a non-contact technique, it can be applied to existing processes without adjusting tool paths.

## Introduction

Metal additive manufacturing (AM) is a widely used layer-by-layer process for rapid prototyping and manufacturing of complex three-dimensional metallic structures^1^. However, the disadvantageous anisotropic tensile and fatigue properties of coarse columnar grain structures of AM parts inhibit the widespread use of AM in the manufacturing industry^2,3^. In typical AM processes, thermal gradients are steeply formed inside small melt pools, leading to strong epitaxial growth of columnar grains along the build direction^2,3^.

Among various metal AM materials, Ti-6Al-4V is the most researched material owing to its excellent applicability in the biomedical and aerospace industries^1^. However, because typical Ti-6Al-4V AM parts have coarse columnar prior-β grains, they exhibit anisotropic tensile properties^1^. Therefore, the promotion of fine equiaxed grains in AM parts has become an important research topic for improving their tensile properties^3^.

Various techniques have been proposed for introducing equiaxed grains in the AM parts. Introducing additional particles to assist active nucleation has been proven to be effective in promoting columnar-to-equiaxed transition, although changes in material composition are inevitable^4–6^. Post-processing techniques, such as inter-pass rolling^7^, machine hammer peening^8^, ultrasonic impact treatment^9^ and laser shock peening^10,11^, have also been proposed. However, because these techniques are applied after the solidification of layers, they require more processing time and might limit the complexity of the AM parts. Ultrasound-assisted AM resolves this problem by delivering high ultrasound energy to the melt pool^12^. However, an ultrasound transducer must be attached to the bottom of the base plate to efficiently deliver sufficient energy to agitate the melt pool. To apply this contact-type technique, implementation problems must be solved, because it is difficult to ensure stable effects on the moving melt pool with its three-dimensional trajectory. Recently, delivering localized ultrasound energy inside the melt pool via intensity-modulated laser irradiation was investigated for in-situ grain refinement^13^. As a proof of concept, the technique was verified on a stainless-steel plate, showing that the intensity-modulated laser can simultaneously perform surface melting and ultrasound generation. Meanwhile, a synchronous induction heating-assisted AM has been proposed recently for in-situ microstructural control. However, the task of stably applying the technique for arbitrary-shaped parts remains^14^.

In this study, we propose a pulsed laser-assisted AM (PLAAM) technique to refine the prior-β grains of Ti-6Al-4V parts during laser-directed energy deposition (DED). A nanosecond pulsed laser was incorporated into a DED system to deliver high pulsed energy to the melt pool during AM. Because PLAAM is an in-situ and non-contact technique that affects the melt pool, it can be applied to the AM of complex objects with arbitrary sizes and shapes. Inspired by the contact-type ultrasound technique^12^ and the well-established pulsed laser effects on liquids^15^, the proposed technique exploits laser-induced shock waves, cavitation, and accelerated Marangoni flow inside the melt pool to create a favorable environment for the formation of a fine equiaxed prior-β grain structure.

We experimentally demonstrate that the parts manufactured by PLAAM have finer and nearly equiaxed prior-β grain structures. In addition, we present a physical explanation of how the application of a pulsed laser can alter the prior β-grain structure of the deposited layers.

## Results

### Pulsed laser-assisted additive manufacturing

**Figure 1:**
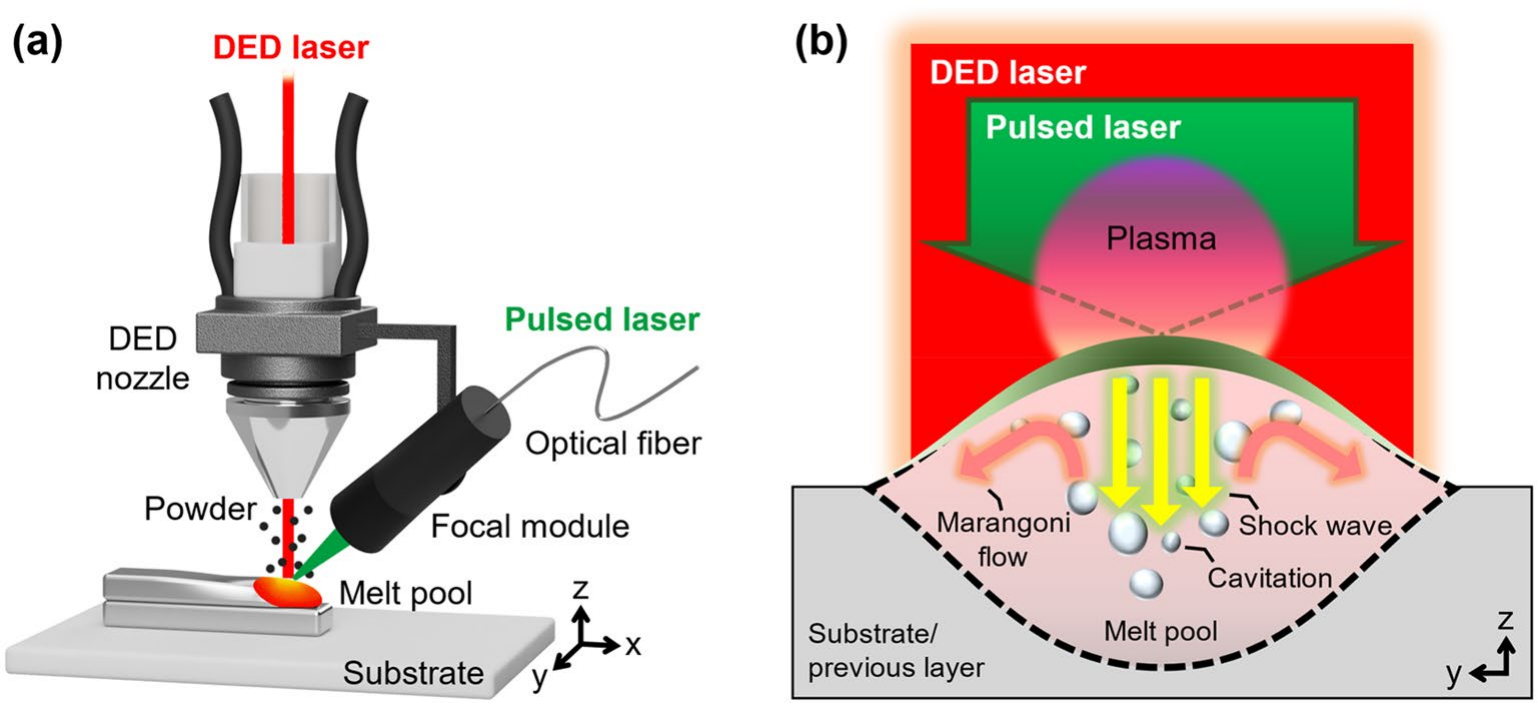
Pulsed laser-assisted AM (PLAAM). (**a**) Off-axis configuration of a PLAAM system. (**b**) The pulsed laser induces shock waves, cavitation, and accelerated Marangoni flow inside the melt pool, providing a favorable environment for grain refinement.

The PLAAM technique is illustrated in Fig. 1. To pinpoint the melt pool and directly deliver the pulsed laser energy during AM, the pulsed laser was focused onto the melt pool using a fiber-guided focal module attached to the DED nozzle. The focal module was fixed to the DED positioning frame so that the foci of the pulsed laser and DED laser coincided during AM. Although an off-axis configuration was adopted in this study, the pulsed laser can be coaxially designed with the DED laser using a dichroic mirror for full integration^16^.

The pulsed laser effects are illustrated in Fig. 1b. The wavelength and pulse duration of the pulsed laser were 532 nm and 10 ns, respectively. The focal size and pulse power density were selected as 2.8 × 10^−3^ cm^2^ and 0.41 GW/cm^2^, respectively, such that shock waves and cavitation could be generated inside the melt pool. With the given power density higher than the dielectric breakdown threshold of titanium, 0.36 GW/cm^2^^17^, an avalanche process of ionization, also known as the dielectric breakdown, occurred in the melt pool with audible ablation sound and bright visible plasma sparks. These phenomena are known to be followed by plasma formation, shock wave propagation, and cavitation generation^15^. In addition, the selected pulsed laser parameter values were sufficient to accelerate the Marangoni flow inside the melt pool^16^. Change in the material composition was expected to be negligible because the laser ablation depth is in the order of the pulsed-laser wavelength (532 nm)^15^. A detailed explanation of these pulsed laser effects on the refinement of the prior-β grains is provided afterwards in the “Discussion” section.

The DED process parameters were selected to maintain a 250 μm target layer height during multilayer AM. With the chosen DED parameters, the energy density was calculated as 100 J/mm^2^, which can assure the total volume fraction of pores below 0.1%^18^. Because the averaged input power increased by the additional puled-laser excitation was only 1.15%, such small extra input power had little effect on the selection of the DED laser power. With a 300-mm/min scanning speed and a 100 Hz repetition rate of the pulsed laser, the distance between the two consecutive pulsed excitations during PLAAM was 50 μm. Because typical Ti-6Al-4V AM samples feature columnar prior-β grains of several millimeters in the building direction and several hundreds of micrometers in the scanning direction^2,12^, the given vertical (250 μm) and lateral (50 μm) excitation intervals were an order of magnitude smaller than the prior-β grain size. Thus, the pulsed laser could effectively alter the prior-β grain structure. Further study on optimizing the pulsed laser parameters is required to maximize grain refinement.

### Prior-β grain refinement

**Figure 2:**
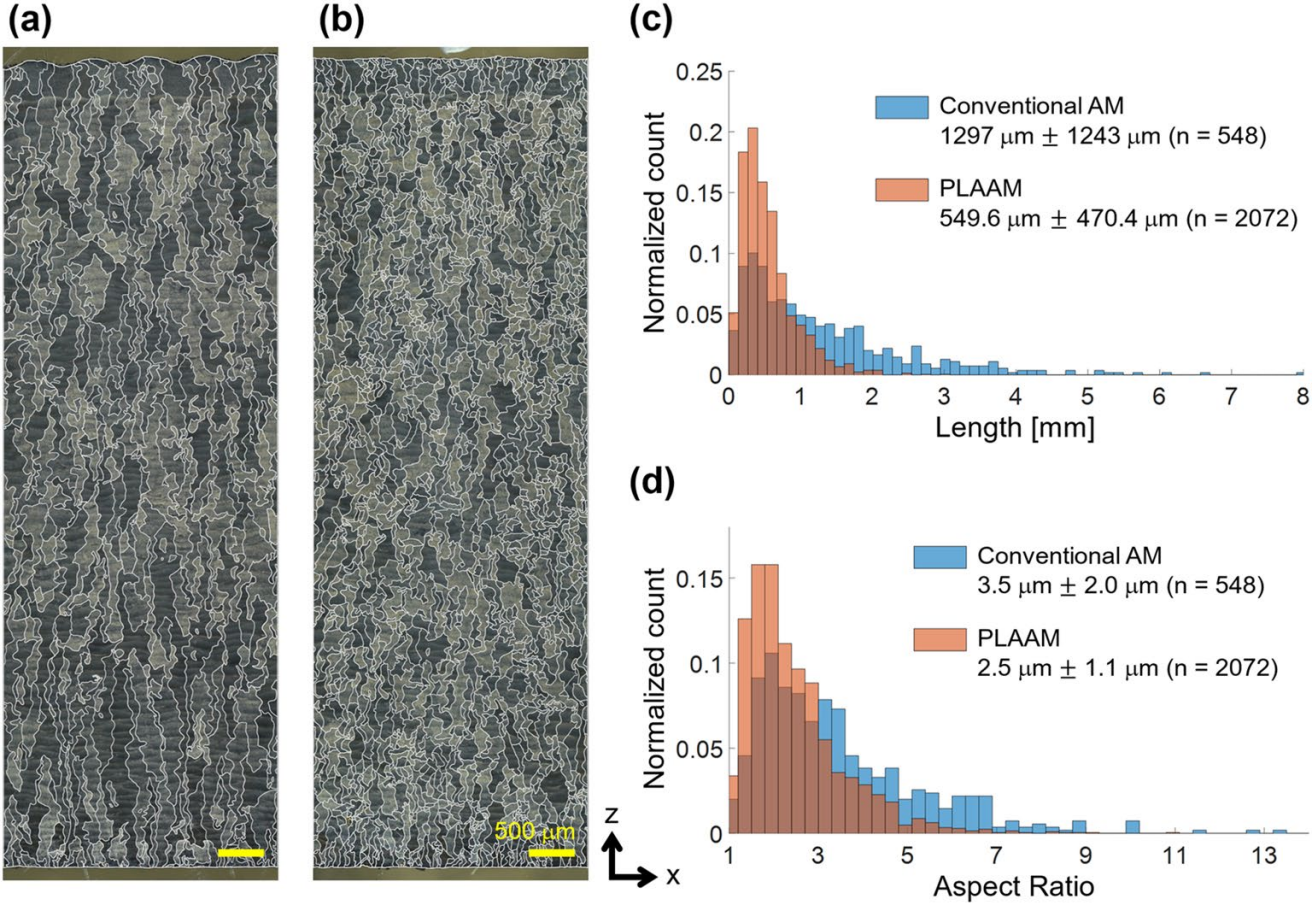
Change in prior-β grain structure. OM images along the build direction of conventional AM (**a**) and PLAAM (**b**) samples. z and x are the build and transverse directions, respectively. Histograms of the length (**c**) and aspect ratio (**d**) of the prior-β grains observed in (**a**) and (**b**). The overlapped histograms are shown in a darker color.

As shown in the optical microscopy (OM) images of Fig. 2a,b, the PLAAM sample exhibited finer and more equiaxed prior-β grains distributed over the entire build height of 30 mm, compared to the conventional AM sample with large columnar prior-β grains. The prior-β grain boundaries were manually traced for further analysis using the ImageJ software^19^. The number of prior-β grains per unit area of the PLAAM sample (6.91 mm^−2^) was 3.78 times that of the conventional AM sample (1.83 mm^−2^), meaning that the PLAAM sample had finer prior-β grains. In addition, the lengths and aspect ratios of the prior-β grains are presented in histograms to statistically show the changes in the grain structure (see Fig. 2c,d). Using the PLAAM technique, the mean length of the prior-β grains decreased from 1297 to 549.6 μm, and the mean aspect ratio decreased from 3.5 to 2.5. Moreover, the variation in the prior-β grain size and shape was smaller using PLAAM than with conventional AM. These results indicate that the PLAAM sample exhibits finer equiaxed prior-β grains than the conventional AM sample.

**Figure 3:**
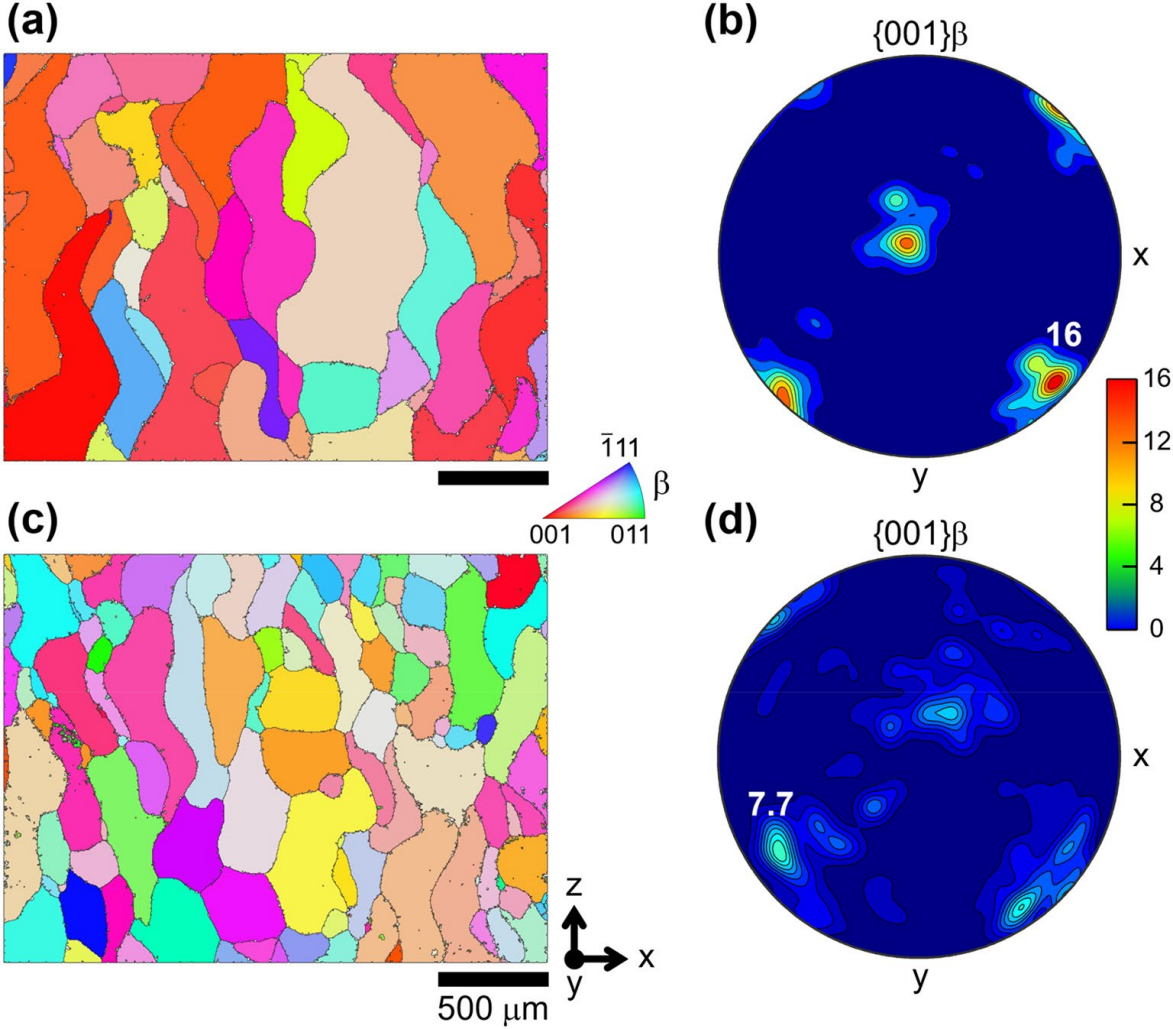
EBSD analysis of the conventional AM (**a**, **b**) and PLAAM (**c**, **d**) samples. Inverse pole figure maps for the β phase along the build direction (**a**, **c**). Contoured pole figures for the β phase (**b**, **d**). z and (x,y) are the build direction and transversal plane, respectively.

For a closer inspection of the prior-β grains, electron backscatter diffraction (EBSD) analysis was conducted on the samples, as shown in Fig. 3. The inverse pole figure maps for the β phase (Fig. 3a,c) show that the PLAAM sample had a nearly equiaxed prior-β grain structure, compared to the conventional AM sample with a columnar prior-β grain structure along the build direction. The inverse pole figure maps were reconstructed using an open-source MATLAB toolbox MTEX^20^. Contoured pole figures for the β phase of the two samples (Fig. 3b,d) were also calculated to quantitatively visualize the texture change with multiples of uniform distribution (MUD) values. The PLAAM sample had a maximum MUD of 7.7, which was less than half that of the conventional AM sample (16). Compared to a strong crystallographic texture in the < 001 > direction shown in the AM sample, a weak texture is observed in the PLAAM sample. These results confirm that the PLAAM sample had a more isotropic structure of finer prior-β grains compared to the conventional AM sample.

## Discussion

The pulsed laser parameters were chosen to (1) accelerate the Marangoni flow in the melt pool by instantaneous and localized heating on the surface^16^; (2) generate shock waves; and (3) create cavitations in the melt pool following dielectric breakdown^15^. Here we discuss how these effects enhance equiaxed nucleation in the melt pool. Equiaxed nucleation can be promoted by increasing constitutional supercooling^21^. To increase constitutional supercooling, either the melting temperature should be increased, or the thermal gradient should be decreased.

The melting temperature can be increased by high-pressure shock waves impinging on the melt pool. Based on the Clausius–Clapyron equation, the relationship between the melting temperature elevation and pressure change can be expressed as follows:

1 $$\frac{{1}}{{{\textit{T}}_{{\text{m}}} }}{\text{d}\textit{T}}_{{\text{m}}} { = }\frac{{\textit{V}}}{{\textit{H}}}{\text{d}\textit{P}},$$

where ${\textit{T}}_{{\text{m}}}$ is the melting temperature (K), ${\textit{P}}$ is the pressure (Pa), ${\textit{V}}$ is the volume change upon melting (m^3^), and ${\textit{H}}$ is the latent heat of fusion (J/kg). Based on Eq. (1) and the material properties of Ti-6Al-4V summarized in Table 1, a shock pressure of 100 MPa produced by cavitation^22^, for example, leads to a melting-temperature increase of 8.66 K. Moreover, with a power density of 0.41 GW/cm^2^, the pulsed-laser-induced shock waves impinged upon the melt pool create pressures up to 336.2 MPa according to Lindl’s Equation^23,24^:

2 $${\textit{P}} = 4 \times 10^{3} \times \left( {\frac{I}{\lambda }} \right)^{2/3} ,$$

where *P* is the generated shock pressure (GPa), *I* is the laser power density (10^15^ W/cm^2^), and λ is the wavelength of the laser (μm). This high pressure leads to a melting-temperature increase of 29.31 K. This increase in the melting temperature is directly translated to the increase in constitutional supercooling^25^. With increased constitutional supercooling, initial nuclei are likely to be activated near the cavitations and shock wave fronts. These events can suppress the epitaxial growth of columnar grains by competition. Previous works have experimentally demonstrated a similar mechanism using ultrasound-induced cavitations to promote equiaxed nucleation in welding^25^ and in AM^12,21^. The performance of PLAAM can be compared to a recent study^26^ about alloying yttria-stabilized zirconia (YSZ) for grain-refined AM of Ti-6Al-4V. The authors showed that adding 5% YSZ to Ti-6Al-4V increased constitutional supercooling by 112 K and decreased the average length of the prior-β grains from > 2 mm to 118 μm.

**Table 1:** Properties of the Ti-6Al-4V alloy^27^.

Property (unit)	Value
Molar mass (g/mol)	446.0697
Solid density (kg/m^3^)	4420 – 0.154 (T – 298 K)
Liquid density (kg/m^3^)	3920 – 0.680 (T – 1923 K)
Liquidus temperature (K)	1923
Latent heat of fusion (J/kg)	2.86 × 10^5^

In PLAAM, the pulsed laser effects further increased constitutional supercooling by decreasing the thermal gradient inside the melt pool. Because of the opposing directions of the Marangoni flow and pulsed laser-induced shock waves as illustrated in Fig. 1b, a vigorous turbulent flow is created inside the melt pool^16^. Moreover, cavitation-induced shock waves generate sporadic and instantaneous turbulence inside the melt pool. In this environment, nuclei activated by increased constitutional supercooling are distributed in the melt pool. Hence, the epitaxial growth of large columnar prior-β grains is further subjected to competition.

## Conclusion

In summary, we have demonstrated in-situ grain refinement of Ti-6Al-4V parts using a hybrid AM technique named as PLAAM. The proposed technique exploits a high-power-density pulsed laser to create a favorable environment for the growth of equiaxed prior-β grains. Because the technique is a non-contact type, it can be applied to any existing AM equipment without adjusting the arbitrary tool path. Microstructural assessments show that the PLAAM sample has smaller and more equiaxed prior-β grains compared to the conventional AM sample having large columnar prior-β grains. Also, the maximum value of MUD of the β phase decreased from 16 to 7.7 when using the PLAAM technique, indicating a weakened texture and refined prior-β grains. A detailed explanation of the pulsed laser effects on the melt pool is also presented for further adaptation of the proposed approach. Because the equiaxed prior-β grain structure is known for its isotropic and high tensile properties, the proposed technique is expected to be extensively studied for producing high-quality metal AM parts.

## Methods

A Q-switched Nd:YAG pulsed laser (Centurion+, Quantel) was incorporated into a laser metal powder DED system (MX-400, InssTek) equipped with a continuous-wave Yb-fiber DED laser, as illustrated in Fig. 1a. The working distances of the pulsed laser and DED laser were 43 mm and 9 mm, respectively. The power and focal diameter of the DED laser, powder feed rate, and flow rate of coaxial argon gas were set to 100 W, 800 μm, 1 g/min, and 6.0 L/min, respectively.

Walls with dimensions of 30 × 30 × 1.3 mm (height × width × thickness) made of 120 layers were fabricated by both the conventional AM and PLAAM techniques. Commercial-grade 23 Ti-6Al-4V powder (AP&C) with a diameter ranging from 45 to 150 μm was used to deposit the layers. The samples were separated from the substrate for post-fabrication grain structure inspection. The samples were polished and etched using a Kroll’s etchant (4 mL of HF, 6 mL of HNO_3_, and 90 mL of H_2_O). The structure and shape of the prior-β grains were examined via OM (Stemi 508, ZEISS). The microstructure texture was examined by EBSD using a detector (QUANTAX EBSD, Bruker) operated with an acceleration voltage of 20 kV and a step size of 4.2 μm.

## Data Availability

The datasets used and/or analyzed during the current study are available from the corresponding author upon reasonable request.
